# Quality of Life in Women Diagnosed with Breast Cancer after a 12-Month Treatment of Lifestyle Modifications

**DOI:** 10.3390/nu13010136

**Published:** 2020-12-31

**Authors:** Concetta Montagnese, Giuseppe Porciello, Sara Vitale, Elvira Palumbo, Anna Crispo, Maria Grimaldi, Ilaria Calabrese, Rosa Pica, Melania Prete, Luca Falzone, Massimo Libra, Serena Cubisino, Luigina Poletto, Valentina Martinuzzo, Sergio Coluccia, Nadia Esindi, Flavia Nocerino, Anita Minopoli, Bruna Grilli, Pasqualina C. Fiorillo, Marco Cuomo, Ernesta Cavalcanti, Guglielmo Thomas, Daniela Cianniello, Monica Pinto, Michelino De Laurentiis, Carmen Pacilio, Massimo Rinaldo, Massimiliano D’Aiuto, Diego Serraino, Samuele Massarut, Laura Caggiari, Chiara Evangelista, Agostino Steffan, Francesca Catalano, Giuseppe L. Banna, Giuseppa Scandurra, Francesco Ferraù, Rosalba Rossello, Giovanna Antonelli, Gennaro Guerra, Amalia Farina, Francesco Messina, Gabriele Riccardi, Davide Gatti, David J. A. Jenkins, Egidio Celentano, Gerardo Botti, Livia S. A. Augustin

**Affiliations:** 1 Epidemiology and Biostatistics Unit, Istituto Nazionale Tumori—IRCCS “Fondazione G. Pascale”, 80131 Napoli, Italy; c.montagnese@istitutotumori.na.it (C.M.); sara.vitale@istitutotumori.na.it (S.V.); elvirapalumbo41@gmail.com (E.P.); a.crispo@istitutotumori.na.it (A.C.); m.grimaldi@istitutotumori.na.it (M.G.); r.pica@istitutotumori.na.it (R.P.); melania.prete.1@hotmail.it (M.P.); lucafk92@hotmail.it (L.F.); sergio.coluccia@hotmail.it (S.C.); f.nocerino@istitutotumori.na.it (F.N.); e.celentano@istitutotumori.na.it (E.C.); l.augustin@istitutotumori.na.it (L.S.A.A.); 2 Department of Clinical Medicine and Surgery, Federico II University, 80131 Napoli, Italy; ilariacalabrese@live.it (I.C.); riccardi@unina.it (G.R.); 3 Department of Biomedical and Biotechnological Sciences Oncologic, Clinical and General Pathology Section, University of Catania, 95131 Catania, Italy; m.libra@unict.it; 4 Cannizzaro Hospital, 95126 Catania, Italy; serena-cubisino@hotmail.it (S.C.); fcatalano1968@tiscali.it (F.C.); giuseppe.banna@medicareonlus.com (C.L.B.); giusy.scandurra@gmail.com (G.S.); 5 Cancer Epidemiology Unit, National Cancer Institute Centro di Riferimento Oncologico, IRCCS, 33081 Aviano, Italy; lpoletto@cro.it (L.P.); nutrizionista.martinuzzo@gmail.com (V.M.); serrainod@cro.it (D.S.); smassarut@cro.it (S.M.); 6 School of Life and Environmental Sciences and Charles Perkins Centre, University of Sydney, Sydney 2006, Australia; nadia_esindi@live.it; 7 Laboratory Medicine Unit, Istituto Nazionale Tumori—IRCCS “Fondazione G. Pascale”, 80131 Napoli, Italy; a.minopoli@istitutotumori.na.it (A.M.); b.grilli@istitutotumori.na.it (B.G.); pasqualina.fiorillo@istitutotumori.na.it (P.C.F.); m.cuomo@istitutotumori.na.it (M.C.); e.cavalcanti@istitutotumori.na.it (E.C.); 8 Clinica Mediterranea, 80122 Napoli, Italy; guglielmo.thomas@outlook.it; 9 Division of Breast Oncology, Istituto Nazionale Tumori—IRCCS “Fondazione G. Pascale”, 80131 Napoli, Italy; d.cianniello@istitutotumori.na.it (D.C.); m.delaurentiis@istitutotumori.na.it (M.D.L.); c.pacilio@istitutotumori.na.it (C.P.); m.rinaldo@istitutotumori.na.it (M.R.); 10 Rehabilitation Medicine Unit, Istituto Nazionale Tumori—IRCCS “Fondazione G. Pascale”, 80131 Napoli, Italy; m.pinto@istitutotumori.na.it; 11 Clinica Villa Fiorita, Aversa 81031, Italy; info@daiuto.it; 12 Immunopathology and Cancer Biomarkers Unit, National Cancer Institute Centro di Riferimento Oncologico, IRCCS, 33081 Aviano, Italy; lcaggiari@cro.it (L.C.); cevangelista@cro.it (C.E.); asteffan@cro.it (A.S.); 13 Ospedale San Vincenzo, 98039 Taormina, Italy; ferrau@oncologiataormina.it (F.F.); rosalbarossello@alice.it (R.R.); gio.antonelli67@gmail.com (G.A.); 14 Ospedale Evangelico Betania, 80147 Napoli, Italy; rino.guerra@live.it (G.G.); amaliafarina@hotmail.it (A.F.); messina52@alice.it (F.M.); 15 Rheumatology Unit, University of Verona, 37129 Verona, Italy; davide.gatti@univr.it; 16 Departments of Nutritional Sciences and Medicine, Temerty Faculty of Medicine, University of Toronto, Toronto, ON M5S, Canada; david.jenkins@utoronto.ca; 17 Clinical Nutrition and Risk Factor Modification Centre, St. Michael’s Hospital, Toronto, ON M5B 1T8, Canada; 18 Li Ka Shing Knowledge Institute, St. Michael’s Hospital, Toronto, ON M5B 1T8, Canada; 19 Scientific Directorate, Istituto Nazionale Tumori—IRCCS “Fondazione G. Pascale”, 80131 Napoli, Italy; g.botti@istitutotumori.na.it

**Keywords:** quality of life, mediterranean diet, breast cancer, lifestyle, physical activity, vitamin D

## Abstract

Healthy lifestyles are associated with better health-related quality of life (HRQoL), favorable prognosis and lower mortality in breast cancer (BC) survivors. We investigated changes in HRQoL after a 12-month lifestyle modification program in 227 BC survivors participating in DEDiCa trial (Mediterranean diet, exercise, vitamin D). HRQoL was evaluated through validated questionnaires: EQ-5D-3L, EORTC-QLQ-C30 and EORTC QLQ-BR23. Baseline changes were tested using analysis of variance. Multiple regression analyses were performed to assess treatment effects on HRQoL. Increases were observed in global health status (*p* < 0.001), physical (*p* = 0.003), role (*p* = 0.002) and social functioning (*p* < 0.001), body image (*p* < 0.001), future perspective (*p* < 0.001), well-being (*p* = 0.001), and reductions in fatigue (*p* < 0.001), nausea and vomiting (*p* = 0.015), dyspnea (*p* = 0.001), constipation (*p* = 0.049), financial problems (*p* = 0.012), sexual functioning (*p* = 0.025), systematic therapy side effects (*p* < 0.001) and breast symptoms (*p* = 0.004). Multiple regression analyses found inverse associations between changes in BMI and global health status (*p* = 0.048) and between serum 25(OH)D levels and breast symptoms (*p* = 0.002). A healthy lifestyle treatment of traditional Mediterranean diet and exercise may impact positively on HRQoL in BC survivors possibly through reductions in body weight while vitamin D sufficiency may improve BC-related symptoms. These findings are relevant to BC survivors whose lower HRQoL negatively affects treatment compliance and disease outcomes.

## 1. Introduction

A global increase in breast cancer (BC) incidence and mortality has been observed in 2018 [1]. Breast cancer (BC) is the most frequently diagnosed cancer in women in most regions of the world and the most frequent cause of death from cancer in 11 regions of the world [2]. BC survival rates are increasing globally reaching 86% at 5 years in Italy [3]. The increase in BC incidence and the high survival rates among BC patients indicate the importance of targeting health-related quality of life (HRQoL) and understanding its relationship with lifestyle including diet and physical activity. Healthy lifestyles have been associated with better quality of life [4] which is in turn associated with favorable prognosis and lower mortality [5,6,7,8,9].

Cancer patients report declines in physical functioning, increased pain and generally reduced quality of life [10,11,12]. This can reduce compliance to oncologic treatment with adverse consequences on cancer prognosis and mortality [13,14]. Diet has been shown to improve quality of life in BC survivors [6,15,16,17,18] and a greater adherence to the Mediterranean diet has been associated with higher physical functioning and health status in women recently diagnosed with BC [4].

Physical activity has also been found effective in improving overall quality of life in BC survivors [19,20] through direct physiologic effects or indirectly by reducing oncologic treatment side effects [8,21].

Few studies have examined the effects of vitamin D supplementation on HRQoL in BC survivors. However, some evidence suggests beneficial effects of vitamin D supplementation on musculoskeletal pain in BC patients with low vitamin D levels and receiving adjuvant anastrozole therapy [22].

Therefore, we investigated HRQoL in BC survivors after a 12-month lifestyle modification program, including higher adherence to the traditional Mediterranean diet, higher physical activity and vitamin D supplementation to reach sufficiency levels.

## 2. Materials and Methods

### 2.1. Study Design

This study is part of an ongoing multicenter randomized controlled trial on the efficacy of a treatment program including dietary modifications, physical activity and vitamin D supplementation (DEDiCa study) on BC relapse [23], approved by the Ministry of Health Italian, Italian Medicines Agency (AIFA) and the ethics committees of each recruiting hospital (ClinicalTrials.gov NCT02786875). Eligible participants were found through surgical lists of participating hospitals. They were contacted by phone and offered to learn more about the study during group information sessions. Informed consent was obtained at baseline from all participants included in the study. Eligible women were randomized to follow either one of two treatments: (a) low glycemic index traditional Mediterranean diet + daily brisk walking + vitamin D supplementation; (b) traditional Mediterranean diet + avoidance of physical inactivity + vitamin D supplementation. Our analyses included 227 participants with complete data at baseline and 12 months. Clinic visits included the evaluation of anthropometric, dietary and biochemical parameters at baseline and at 12 months. Furthermore, through specific questionnaires we obtained information at the baseline and at 12 months on adherence to the Mediterranean diet and on the health-related quality of life (HRQoL). Details of these questionnaires are explained below.

### 2.2. Anthropometric and Physical Activity Level Measurements

Anthropometric data of the participants were collected by the study staff at baseline and 12 months, specifically weight was measured to the nearest 0.5 Kg using a Seca scale (Seca 761) while height to the nearest 1 cm using a Seca stadiometer. Body mass index (BMI) was calculated using the formula weight (kg)/height (m^2^).

Physical activity level was assessing using a step counter (Omron Walking Style IV) provided by study staff prior to the baseline visit. Participants wore the step counter for at least 7 days before each visit. Four categories of activity were identified: sedentary (<5000 steps/day), low activity (5000 < 7500 steps/day), medium activity (7500 < 10,000 steps/day) and high activity (≥10,000 steps/day) [24].

### 2.3. Adherence to Mediterranean Diet

At baseline and 12-month follow-up all participants completed a 7-day food record and the 14-item questionnaire administered by study staff. The 14-point Mediterranean Diet Adherence Screener (MEDAS), developed to control for compliance with the dietary intervention of the study Prevención con Dieta Mediterránea (PREDIMED) [25], was used to assess dietary adherence in people living in a Mediterranean area [26]. MEDAS questionnaire includes 14 questions: 12 investigate the frequency and quantity of foods (olive oil, vegetables, fruit, red or processed meats, butter, soda drinks, legumes, fish, commercial sweets, nuts, wine, sofrito sauce), while two questions are related to the preference of olive oil and meat consumption. Each question has two possible answers and scores; 1 score for “yes” and 0 for “no”. The 14-point MEDAS can range from 0 to 14 where 14 represents the highest adherence to Mediterranean diet.

### 2.4. Dietary Assessment

Dietary data, including beverage intakes and alcohol consumption, were collected by a 7-day food records at baseline and 12-month follow-up. During clinic visits patients returned the food records to the nutritionist who asked supplemental questions where necessary. Data were stored and processed using a professional software (WinFood©) which utilized the CREA—Alimenti e Nutrizione nutritional database. Dietary fiber was calculated as g/1000 kcal, while saturated fatty acids (SFA) and monounsaturated fatty acids (MUFA) as percent of daily energy intake at baseline and 12-month follow-up.

### 2.5. Health Related Quality of Life (HRQoL)

To assess the quality of life at baseline and 12-months, the patients completed three validated questionnaires: the European Quality of Life 5 Dimensions 3 Level (EQ-5D-3L) [27], the European Organization for Research and Treatment of Cancer Quality of Life Questionnaire Core 30 items (EORTC QLQ-C30) and Breast Cancer 23 items (EORTC QLQ-BR23) [28]. The EQ-5D-3L is one of most commonly used generic health-related quality-of-life questionnaire, made by the Euroqol group. The questionnaire comprises five dimensions (mobility, self-care, usual activities, pain or discomfort, and anxiety or depression) and three levels of perceived problems (no problems, some problems and extreme problems). Another part of the questionnaire includes a visual analogue scale (EQ-VAS) that measures patient self-perceived health status. We have used the Italian health states value set [29] to obtain a unique health index score (EQ-5D-index), where the value of 1 represents the best health status and below 1 worse health status; in addition, EQ-VAS evaluates the participant’s self-reported health state on a scale from 0 (worse health status) to 100 best health status.

The EORTC QLQ-C30 (Questionnaire for Quality of Life Assessment in patients with cancer, version 3.0) is a tool for assessing quality of life in cancer patients, consisting of 30 questions subsequently transformed in 15 scales: five functional dimensions (physical, role, emotional, cognitive, and social), three symptom items (fatigue, nausea or vomiting, and pain), six single items (dyspnea, sleep disturbance, appetite loss, constipation, diarrhea, and financial impact) and a global health status/QoL scale [28]. The EORTC QLQ-BR23 (Quality of Life Questionnaire—Breast Cancer) assesses quality of life specifically in breast cancer patients and comprises of 23 questions that evaluate body image, sexual functioning, sexual enjoyment, future perspective, systemic therapy side effects, breast symptoms, arm symptoms and distress from hair loss. For each question there are four answers (not at all, a little, quite a bit, very much). All items are linearly transformed to a 0–100 scale according to a standardized process described in EORTC QLQ-C30 Scoring Manual 3rd Edition [30]. Higher scores for functioning and for global health status indicate better health. Conversely, higher scores for symptoms indicate worse health.

In our analyses we did not include the answers to the questions related to sexual enjoyment and feeling towards hair loss because at least 50% of participants did not respond to these questions at baseline.

### 2.6. Biochemical Measurements

Blood samples were collected at baseline and after 12 months. Serum 25(OH)D concentrations were measured using chemiluminescent immunoassay (CLIA) technology for the quantitative determination of 25-hydroxyvitamin D and other hydroxylated vitamin D metabolites in human serum (LIAISON^®^ 25 OH Vitamin D TOTAL Assay) at the Laboratory Medicine Unit. All analytes were measured in the coordinating hospital routine analytical laboratory (Laboratory Medicine Unit, Istituto Nazionale Tumori—IRCCS “Fondazione G. Pascale”, Napoli, Italy) after quality control procedures.

### 2.7. Statistical Analyses

The present analysis includes data from the first 227 women enrolled in DEDiCa study with complete data at baseline and 12 months. Baseline characteristics were described as numbers (n) and percentages (%). Sociodemographic and clinical characteristics were summarized using tabulations for categorical variables and means and standard deviations (SD) for continuous variables. Means and SD were calculated for all HRQoL dimensions, as well as anthropometric parameters, step count, 25(OH)D levels, dietary measurement at baseline and at 12-month follow-up and changes from baseline were compared with the analysis of variance (ANOVA). These analyses were also performed in strata of BMI at baseline (<25 kg/m^2^ vs. ≥25 kg/m^2^) and in strata of hormone therapy use at 12 months (treatment vs no treatment). Considering there were no significant differences in HRQoL between randomization arms at 12 months, data from both groups were analyzed together. Multiple linear regression models adjusted for terms of age, civil status, education, time from surgery, BMI, step count, 25(OH)D level, MEDAS score, dietary fiber intake, monounsaturated fatty acids (MUFA) and saturated fatty acids (SFA) intakes were performed to assess the association between changes in HRQoL dimensions and selected covariates. The dependent variables were changes in the HRQoL domains and the independent variables were the lifestyle treatment components at baseline, 12 months and their time changes. The dependent variables were chosen based on significant ANOVA variation from baseline to 12 months. Results are reported as estimates beta coefficients, *p* value and R^2^ adjusted for the goodness of the model. The Statistical Package for the Social Sciences (SPSS) software, version 26.0 (Chicago, IL, USA) was used for all data analyses. Results were considered statistically significant at *p*-value < 0.05.

## 3. Results

Baseline characteristics of randomized participants (*n* = 227) are shown in Table 1. Mean age (±SD) was 52.3 ± 9.3 years (45% < 50 years), mean body mass index (BMI) was 27.3 kg/m^2^ ± 5.8 (43% normal weight, 31% overweight and 26% obese), 52.4% non-smokers, 76.3% were inactive and 62.6% showed adherence to the Mediterranean diet. The majority of participants, 96% were postmenopausal (natural or pharmacologically induced), 81.1% were married or common-law and 65.6% attained high school or higher education. Moreover, Table 1 shows cancer characteristics including staging, molecular subtypes and oncologic treatment. Patients undertaking hormone therapy were 54.6% while 16% were still receiving adjuvant chemotherapy at baseline. Participants who reported at least one comorbidity at baseline were 41%: 5.7% reported type 2 diabetes, 23.3% hypertension and 22.9% hypercholesterolemia.

**Figure 1:**
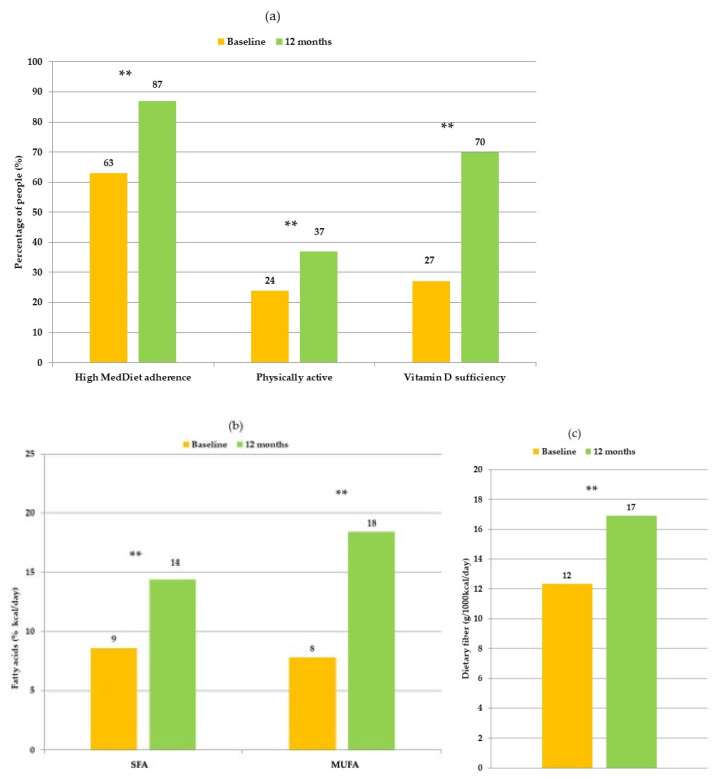
(**a**) Comparison of participants with high Mediterranean diet adherence (MEDAS score > 7), physically active (>7500 steps/day) and with vitamin D sufficiency (≥30 ng/mL) at baseline and at 12 months; (**b**) Comparison of participants’ fatty acids and (**c**) dietary fibers intakes at baseline and 12 months. (**) significance *p* < 0.001.

After 12 months, most participants showed high adherence to lifestyle treatment resulting in increased Mediterranean diet adherence, higher intakes of dietary fiber and MUFA and lower intakes of SFA, increased physical activity and circulating vitamin D levels increased reached sufficiency (≥30 ng/mL) in 70% of participants (Figure 1a–c).

**Figure 2:**
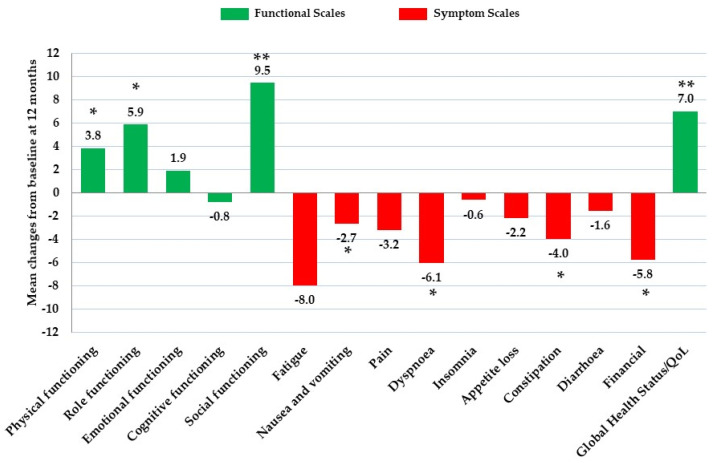
Mean changes of EORTC QLQ-C30 (European Organization for Research and Treatment of Cancer Quality of Life Questionnaire Core 30) scores from baseline at 12 months. (*) significance *p* < 0.05; (**) significance *p* < 0.001.

In the EORTC QLQ-C30 questionnaire, compared to baseline, at 12 months we observed that patients had significantly higher scores for physical functioning (*p* = 0.003), role functioning (*p* = 0.002), and social functioning (*p* < 0.001); among symptoms, at 12 months patients had significantly lower scores for fatigue (*p* < 0.001), nausea and vomiting (*p* = 0.015), dyspnea (*p* = 0.001), constipation (*p* = 0.049) and financial difficulties (*p* = 0.012). At 12 months general global health status significantly increased (*p* < 0.001) compared to baseline, indicating an improved HRQoL (Table 2, Figure 2). Pain symptom scores were also reduced at 12 months albeit not significantly (*p* = 0.099).

**Figure 3:**
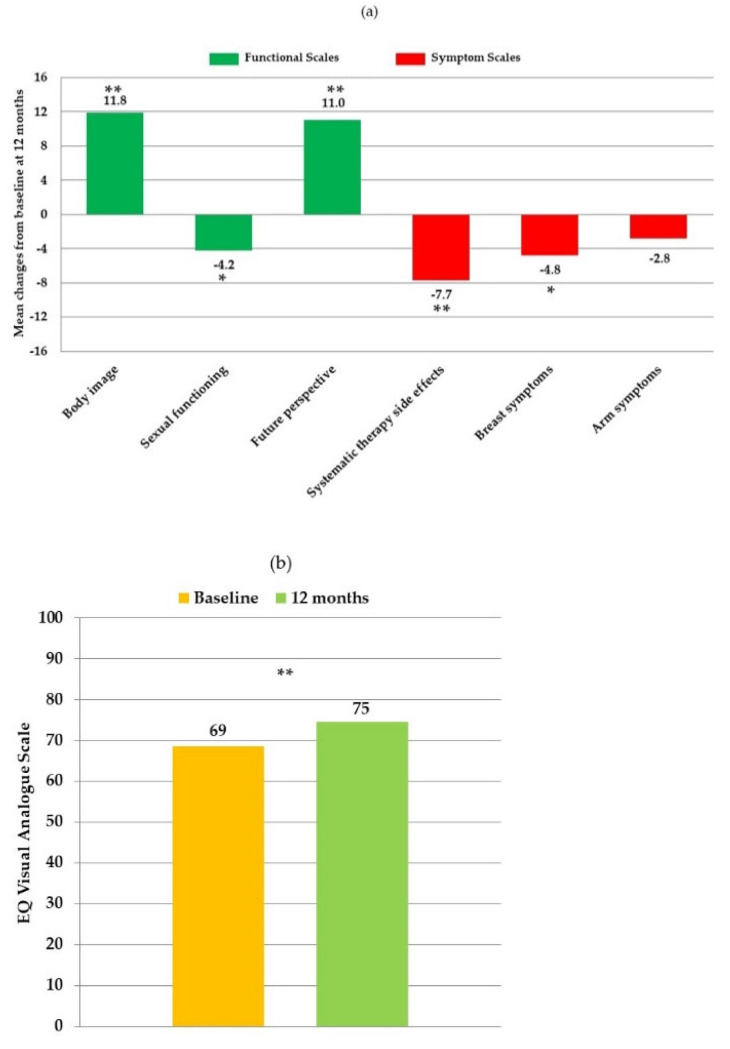
(**a**) Mean changes of European ORTC QLQ-BR23 (European Organization for Research and Treatment of Cancer Quality of Life Questionnaire Breast 23) scores of participants. (**b**) Comparison of participants EQ VAS (European Quality of Life Visual Analogue Scale) at baseline and at 12 months. (*) significance *p* < 0.05; (**) significance *p* < 0.001.

In the breast cancer specific questionnaire EORTC QLQ-BR23, at 12 months participants had significantly higher scores for body image (*p* < 0.001) and future perspective (*p* < 0.001) but lower scores for sexual functioning (*p* = 0.025). Among symptoms, at 12 months patients had significantly lower scores for systematic therapy side effects (*p* < 0.001) and breast symptoms (*p* = 0.004) (Table 2, Figure 3a). Reductions in arm symptom scores were also observed albeit not significantly (*p* = 0.104). The EQ-5D-3L questionnaire showed significantly higher scores for the visual analogue scale (VAS) (74.6 vs. 68.6; *p* = 0.001) at 12 months, indicating improved overall wellbeing (Table 2, Figure 3b).

When data were stratified by baseline BMI, changes in scores for HRQoL components did not differ significantly at 12 months compared to baseline, except for EQ-5D-3L index scores which significantly increased in normal weight patients indicating improved health status and decreased in overweight/obese (0.034 ± 0.077 vs. −0.013 ± 0.094; *p* < 0.001, respectively). When stratifying data by hormone therapy use at 12 months, we observed significantly higher scores for body image in patients not taking hormone therapy compared to those taking hormone therapy (18.0 ± 20.8 vs. 10.0 ± 24.6; *p* = 0.038, respectively; data not shown) while reductions in sexual functioning were significantly higher in patients not taking hormone therapy compared to those taking hormone therapy (−9.7 ± 22.8 vs. −2.6 ± 20.2; *p* =0.039, respectively; data not shown).

In multivariate analyses relevant changes (β > 0.20; *p* < 0.05) were reported only for breast symptoms changes which were inversely related to changes in serum 25(OH)D levels.

## 4. Discussion

Quality of life improved in women recently diagnosed with BC undertaking oncologic and lifestyle treatments. Compared to baseline, increases were found in global health status, self-perceived health status and functional scales, in most domains, while decreases were found in symptom scales for most items, after 12 months. Specifically, we observed improvements in physical functioning, role functioning and social functioning, and reductions in fatigue, nausea and vomiting, dyspnea, constipation and financial difficulties.

We found that scores improved in functional scales for body image and future prospective, and in systematic therapy side effects and breast symptoms, after 12 months of lifestyle modifications. The increased adherence to the Mediterranean diet measured by MEDAS questionnaire and by increased dietary fiber and MUFA with reduced SFA intakes, by the increased step count and by the normalization of vitamin D levels, confirm that lifestyle modifications were implemented.

Although our multivariate analyses did not show that many of the above treatment components singularly explained changes in HRQoL components (except for vitamin D) the model indicated that changes in BMI were inversely related to changes in global health status indicating improved HRQoL in those who lost weight. This finding has been confirmed by others [31]. Among women who completed the 12-month treatment, two-thirds were overweight/obese at baseline and showed an average weight loss of 1.9 kg despite the majority were taking oncological hormone therapy which is known to increase body weight [32]. Weight loss in a lifestyle modification trial could be due to dietary and exercise modifications which is in line with our findings of significantly improved diet quality and physical activity after 12 months.

We had previously demonstrated that higher adherence to the Mediterranean diet in BC survivors was associated with higher HRQoL, particularly for physical functioning, sleep, pain and overall well-being [4]. Generally, a healthy dietary pattern is associated with better HRQoL [17,18,33,34] including fatigue which is prevalent in BC survivors and is associated with shorter overall survival [35]. Dietary intervention rich in fruits, vegetables, whole grains, and omega-3 fatty acid-rich foods, which are staple foods of the Mediterranean diet, significantly decreased cancer-related fatigue and improved sleep quality in BC survivors [34]. A possible mechanism for the relationship between a healthy diet and HRQoL may be through inflammation [36,37] and the Mediterranean diet has been previously associated with reduced inflammation [38]. Considering the increased life expectancy of BC survivors, adherence to a healthy diet may offer multiple health benefits including reduced cardiovascular disease risk, the most common comorbidity and cause of death in BC survivors [9] which can also negatively impact on quality of life. Furthermore, a higher adherence to the Mediterranean diet has been shown to reduce the risk of chronic diseases and overall mortality [39,40].

Physical exercise is another lifestyle component that can positively influence cancer outcomes and treatment-related side effects, quality of life, BC recurrence, and overall survival in BC patients [8]. During our 12-month lifestyle treatment participants increased physical activity level and the number of physically active patients increased from 54 to 86 while the number of sedentary patients decreased from 104 to 71 after 12 months. Physical activity is one of the mainstays of cancer prevention and it is also included in guidelines for BC survivors [41]. Higher physical activity was related to better quality of life in BC patients shortly after adjuvant treatments [42] and it positively influenced cancer- and treatment-related side effects including fatigue and peripheral neuropathy as well as aromatase inhibitor-related arthralgia [8,20,42,43].

Vitamin D may be another treatment component in our study influencing HRQoL. In our study we found significant increases in circulating vitamin D after 12-month supplementation, with 70% of patients reaching sufficiency from 27% at baseline. Multivariate analyses indicated that increased serum vitamin D levels were indeed associated with lower scores for systematic therapy side effects. Although we did not find a significant reduction in pain, there was a trend towards less pain severity at 12 months, although worsening of pain symptoms was expected over time due to side effects of oncologic hormonal therapy (e.g., tamoxifen and aromatase inhibitors) considering the higher number of patients taking hormone therapy at 12-month (77%) compared to baseline (55%). Vitamin D is known to exert a wide range of health effects, including those relating to musculoskeletal structure. Considering the high prevalence of vitamin D deficiency in BC survivors, it is likely that suboptimal vitamin D levels may contribute to the increased incidence and severity of musculoskeletal symptoms experienced by aromatase inhibitor-treated women. It has been reported that non-deficient vitamin D levels and vitamin D supplement use were associated with higher self-reported quality of life in BC survivors [44]. Other studies found beneficial effects of vitamin D supplementation on musculoskeletal pain in BC patients receiving aromatase inhibitor therapy [22,45,46]. Among adverse effects of aromatase inhibitors, pain has a negative impact on quality of life, on treatment compliance and on survival of BC patients [47,48,49,50]. A possible mechanism for the beneficial effect of vitamin D arthralgia and myalgia may be its anti-inflammatory activity.

The present study has some strengths and limitations. Data derives from a multicenter clinical trial where participants were closely followed with quarterly visits, all measurements were collected from centrally trained research staff and all analyses were performed in a central research location. This allowed to collect a large number of covariates which were used in our analyses. Limitations include HRQoL as secondary end point of DEDiCa trial which has a different aim [23] and we did not compare the intervention to the control group for the purpose of the current analysis. Patients were enrolled at different stages of cancer treatment which may have differently affected HRQoL. Although response-shift may affect quality of life outcomes [51] our study included patients enrolled within one year of surgery. At baseline only 16% of participants in the study were still receiving chemotherapy, 50% completed chemotherapy treatment and 34% did not receive chemotherapy treatment, therefore it is unlikely that chemotherapy influenced HRQoL significantly. When we repeated analysis stratifying patients by hormone therapy use at 12 months we found similar results for most components of the HRQoL questionnaires. Finally, we repeated analysis stratifying patients by BMI at baseline and we found similar results for most components of the HRQoL questionnaires, except for EQ-5D-3L index scores (mobility, self-care, usual activities, pain/discomfort and anxiety/depression) where higher scores were seen in normal weight patients and lower scores in overweight and obese patients. Finally, reporting findings at 12 months, instead of 24 or 36 months, may decrease the likelihood of significant HRQoL changes due to lifestyle modifications which may be shadowed by the strong therapy side effects in the first year which tend to subside afterwards.

## 5. Conclusions

To date, there are few studies evaluating the effects of a combined lifestyle program on HRQoL in BC survivors. Our study analyzed and reported in details quality of life aspects in BC patients after a lifestyle program of dietary modification, daily walking and vitamin D supplementation. In conclusion, a healthy lifestyle modification of traditional Mediterranean diet and exercise may impact positively on HRQoL in BC survivors possibly through reductions in body weight while correcting vitamin D sufficiency may improve BC-related symptoms. These findings are relevant to BC survivors whose lower HRQoL negatively affects treatment compliance and disease outcomes. Future studies on BC survivors should be carried out considering these findings to determine the relationship between lifestyle factors and HRQoL in BC survivors and to investigate mechanisms.

## Figures and Tables

**Table 1:** Baseline characteristics of the participants (*n* = 227).

	*n*	(%)
**Age (years)**		
<50 yrs	101	44.5
≥50 yrs	126	55.5
**Education (years of school)**		
≤11 yrs	78	34.4
≥12 yrs	149	65.6
**Civil status ** ^**a**^		
Single ^c^	43	18.9
Married (or common law)	184	81.1
**Menopausal status**		
Menopause	217	95.6
Premenopause	10	4.4
**Smoking status**		
Non-smoker	119	52.4
Smoker	40	17.6
Former smoker	68	30.0
**Body Mass Index (kg/m** ^**2**^ **) ** ^**b**^		
Normal weight	97	42.7
Overweight/Obese	129	57.3
**Physical activity (steps/day) ** ^**c**^		
Sedentary	105	46.3
Low active	68	30.0
Somewhat active	37	16.3
Active	17	7.5
**Mediterranean diet adherence **		
Low (≤7))	85	37.4
High (>7)	142	62.6
**Number of comorbidities ** ^**d**^		
0	134	59.0
1	59	26.0
>1	34	15.0
**Time from surgery**		
<8 months	105	46.3
≥8 months	122	53.7
**Molecular subtype**		
Luminal A	123	54.2
Luminal B	26	11.5
HER2+	42	18.5
Triple negative	36	15.9
**Cancer Stage ** ^**e**^		
I	67	29.5
II	128	56.4
III	32	14.1
**Cancer treatment**		
Adjuvant chemotherapy		
Never	78	34.4
Not current	112	49.3
Current	37	16.3
Radiotherapy		
Never	98	43.2
Not current	112	49.3
Current	17	7.5
Biological therapy		
Never	197	86.8
Not current	1	0.4
Current	29	12.8
Hormone therapy		
No	103	45.4
Yes (current)	124	54.6

^a^ Single are widow, divorced or maiden; ^b^ Normal weight < 25.0 kg/m^2^, Overweight 25.0–29.9 kg/m^2^, Obese ≥ 30.0 kg/m^2^; ^c^ Physical activity (steps/day): Sedentary (<5000), Low active (5000–7499), Somewhat active (7500–9999), Active (≥10,000); ^d^ Type 2 Diabetes, Hypertension, Hypertriglyceridemia, Hypercholesterolemia; ^e^ based on the TNM system (T, size of primary tumor; N, lymph nodes involved; M, metastasis).

**Table 2:** Comparison of European Organization for Research and Treatment of Cancer Quality of Life Questionnaire Core 30 items (EORTC QLQ-C30), Breast Cancer 23 items (EORTC QLQ-BR23), and European Quality of Life 5 Dimensions 3 Level (EQ 5D 3L) scores of participants at baseline and at 12 months.

	Baseline			12 Months			Changes			
**EORTC QLQ-C30**	**N**	**Mean**	**SD**	**N**	**Mean**	**SD**	**N**	**Mean**	**SD**	***p***
**Functioning**										
Physical functioning	226	83.16	14.04	214	86.95	12.48	213	3.8	10.0	**0.003**
Role functioning	224	80.88	21.99	212	86.79	17.58	210	5.9	21.3	**0.002**
Emotional functioning	226	76.13	20.59	215	78.33	18.96	214	1.9	19.2	0.239
Cognitive functioning	225	82.37	21.14	213	81.77	18.25	211	−0.8	17.9	0.750
Social functioning	226	78.25	24.84	216	88.12	17.02	215	9.5	22.9	**<0.001**
Symptoms										
Fatigue	227	31.68	22.28	213	24.15	18.00	213	−8.0	21.9	**<0.001**
Nausea and vomiting	226	7.15	12.93	214	4.36	10.79	213	−2.7	12.9	**0.015**
Pain	226	22.58	21.42	213	19.33	19.51	212	−3.2	19.5	0.099
Dyspnoea	226	18.73	22.40	215	12.25	19.06	214	−6.1	23.5	**0.001**
Insomnia	226	28.17	27.36	211	27.01	26.87	210	−0.6	28.0	0.656
Appetite loss	227	7.20	17.24	216	4.94	13.50	216	−2.2	19.7	0.127
Constipation	200	15.00	22.85	208	10.74	20.65	185	−4.0	24.7	**0.049**
Diarrhoea	225	8.15	16.30	216	6.48	14.70	215	−1.6	19.3	0.261
Financial	224	16.96	25.25	216	11.42	20.67	213	−5.8	24.3	**0.012**
**Global Health Status/QoL**	225	63.89	21.30	216	70.87	17.90	214	7.0	20.4	**<0.001**
**EORTC QLQ-BR23**										
**Functioning**										
Body image	226	65.04	29.98	215	77.17	23.39	214	11.8	24.0	**<0.001**
Sexual functioning	222	81.83	22.09	213	77.0	22.73	208	−4.2	21.0	**0.025**
Future perspective	226	45.58	34.61	216	57.10	30.00	215	11.0	29.3	**<0.001**
**Symptoms**										
Systematic therapy side effects	225	24.06	18.35	214	16.07	11.60	213	−7.7	15.4	**<0.001**
Breast symptoms	224	20.65	18.71	215	15.97	15.12	212	−4.8	18.8	**0.004**
Arm symptoms	224	20.39	19.16	214	17.44	18.59	211	−2.8	17.0	0.104
**EQ-5D-3L**										
EQ INDEX SCORE	225	0.88	0.10	219	0.88	0.11	218	0.01	0.09	0.237
EQ VAS SCORE	222	68.61	15.91	222	74.55	15.30	218	5.80	14.82	**0.001**

SD: standard deviation; Changes: mean changes from baseline to 12 months; QoL: quality of life; VAS: visual analogue scale. Bold data indicate statistically significant *p*-values (significance *p* < 0.05).

## Data Availability

The data presented in this study are available on request from the corresponding author.
